# Neonatal hand, foot, and mouth disease due to coxsackievirus A6 in Shanghai

**DOI:** 10.1186/s12887-020-02262-y

**Published:** 2020-08-03

**Authors:** Shanshan Xu, Huajun Li, Peng Qiao, Guofeng Xu, Dongying Zhao, Xiaoyan Lin, Yu Qin, Huiju Yu, Xi Zhang, Wanju Zhang, Lisu Huang

**Affiliations:** 1 Department of Pediatric Infectious Diseases, Xinhua Hospital, Affiliated to Shanghai Jiao Tong University School of Medicine, Shanghai, 200092 China; 2 Department of Infectious Disease Control, Yangpu District Centers for Disease Control and Prevention, Shanghai, 200093 China; 3 Department of Pediatric Surgery, Xinhua Hospital, Affiliated to Shanghai Jiao Tong University School of Medicine, Shanghai, 200092 China; 4 Department of Neonatology, Xinhua Hospital, Affiliated to Shanghai Jiao Tong University School of Medicine, Shanghai, 200092 China; 5 Department of Pediatric Internal Medicine, Hangzhou Children’s Hospital, Hangzhou, 310000 Zhejiang Province China; 6 Department of Pediatric Internal Medicine, Xingtai People’s Hospital, Xingtai, 054001 Hebei Province China; 7 Clinical Research Unit, Xinhua Hospital, Affiliated to Shanghai Jiao Tong University School of Medicine, Shanghai, 200092 China; 8 Pathogen Diagnosis and Biosafety Department, Shanghai Public Health Clinical Center, Fudan University, Shanghai, 201508 China

**Keywords:** Hand-foot-and-mouth disease, Neonate, Coxsackievirus A6, Clinical symptom, Transmission route, Immunologic function

## Abstract

**Background:**

Evidence of hand, foot, and mouth disease (HFMD) in neonates is limited. The aim of this study was to evaluate the clinical symptoms, pathogens, possible transmission routes, and prognosis of neonatal HFMD in Shanghai.

**Methods:**

This was a case-control study based on the HFMD registry surveillance system. All neonates and infected family members were enrolled between 2016 and 2017 in Shanghai. Neonates with HFMD were followed for at least half a year. Detailed questionnaires, medical history, and physical examination were recorded. Routine blood examination, liver and renal function, immunophenotypes of peripheral blood lymphocytes (CD3, CD4, and CD8 T-cells; NK cells), immunoglobulin (Ig) M, IgG, and IgA, and cytokine interleukin (IL-1β, IL-2R, IL-6, IL-8, IL-10, and TNF-α) levels were measured. All rectal swab specimens were collected and genotyped for enterovirus, and phylogenetic analysis based on the VP1 sequences of coxsackievirus A6 (CV-A6) was performed to investigate molecular and evolutionary characteristics. *T*-test or nonparametric test was used to evaluate the differences. Logistic analysis was applied to calculate the risk of clinical manifestations in the group of HFMD neonates and their paired siblings.

**Results:**

There were 16 neonates among the 12,608 diagnosed patients with HFMD, accounting for 0.13%. All neonatal infections were transmitted by other members of the family, mainly the elder siblings, and were caused by CV-A6. CV-A6 was the emerging and predominant causative agent of HFMD in Shanghai. None of the neonates with HFMD experienced fever, onychomadesis, or severe complications. However, two elder sibling patients showed lethargy, and one developed hypoperfusion. In the elder siblings with HFMD, the proportion of white blood cells was generally higher than in neonates with HFMD. The immunologic function of the neonates with HFMD was basically normal. The levels of inflammatory markers were higher in both neonates and elder siblings with HFMD compared to age-matched controls. The clinical symptoms receded about 1 week after onset. None of the neonates had sequelae.

**Conclusions:**

In our study, CV-A6 infection in neonates was benign, but had the character of family clustering. Due to the two-child policy in China, elder siblings may be the main route of HFMD transmission.

## Introduction

Hand, foot, and mouth disease (HFMD) is a common acute enterovirus (EV) infection, characterized by short-lasting fever, mouth ulcers, and vesicles on the hands, feet, or hips [1]. In March 2008, a sudden outbreak of HFMD occurred in Anhui Province, China. In May, HFMD was defined as a C-class notifiable disease. HFMD has the highest incidence among communicable diseases since 2009 and has become an important public issue [2, 3]. Although HFMD is generally a mild clinical syndrome [4, 5], serious complications may occur [6]. Although HFMD age of onset is widely variable, ranging from neonatal age to 70 years, children aged 5 years and younger are the most susceptible subjects and may develop severe clinical symptoms [7, 8]. Reportedly, subjects younger than 3 years have an increased risk of severe HFMD [2, 8]. However, to date, the age-specific risk of severe HFMD in young children has not been established.

Human EVs belong to the family Picornaviridae, and based on the degree of their genetic relatedness, comprise four species, EV-A to D. Among them, the serotypes EV-A71, Coxsackievirus A16 (CV-A16), CV-A6, and CV-A10, which are frequently associated with HFMD, belong to the EV-A species [9]. CV-A16 and EV-A71 are responsible for most of the large outbreaks [10]. Among healthy individuals in Shanghai, 50.5 and 54.2% are positive for neutralising antibodies against EV-A71 and CV-A16, respectively [11]. Beginning in 2008, CV-A6 has been increasingly reported as a cause of HFMD outbreaks worldwide, and it may be associated with more severe diseases than typical HFMD [4, 12–18].

HFMD can be transmitted both horizontally (faecal-oral/respiratory route) and vertically (prenatal infection). Most new-borns presenting with serious EV disease acquire the infection from a symptomatic mother in the perinatal period; up to 60% of the mothers of infected infants report febrile illness during the last week of pregnancy [19]. Additionally, serious EV disease may be acquired through nosocomial transmission, spreading throughout nurseries via caregivers engaged in mouth care, gavage feeding, and other activities requiring direct contact. Close contact with infected family members may be also an important route of transmission.

In this prospective cohort study, the neonates with HFMD and their families were recruited in Shanghai in 2016–2017, and the epidemiological features, clinical presentation, pathogens, genes, and immune function were compared in neonates with HFMD and their diseased siblings.

## Materials and methods

### Participants and specimens

This was a case-control study based on the National Registry of HFMD. The Chinese government established a network-based national surveillance system for HFMD since 2009. In Shanghai, local health providers and physicians are required to report clinically diagnosed HFMD cases to the Shanghai Municipal Centre for Disease Control and Prevention (CDC) within 24 h via the surveillance system. Basic epidemiologic and clinical information is recorded for each HFMD patient [20]. Sixteen local CDCs, representing as many districts, are responsible for sample collection and transport. The specimens of patients were sampled for pathogen testing at local sentinel hospitals in each district. At least ten outpatients were diagnosed with HFMD each month. The clinicians could also test the specimens as the conditions required. Throat and/or faecal swabs were sent directly to microbiology laboratories at the local CDCs, where the presence of EV-A71, CV-A16, CV-A6, CV-A10, and other EVs was confirmed by real time RT-PCR [11]. The vast majority of children with HFMD are treated in two designated hospitals, the Children’s Hospital of Fudan University and the Xinhua Hospital affiliated to Shanghai Jiao Tong University School of Medicine.

All cases were diagnosed according to the criteria specified by the HFMD Prevention and Treatment Guidelines [21]. Patients who had a rash, with or without fever, and no other organ damage, were classified as having common HFMD. Those with any complication (i.e., aseptic meningitis, brainstem encephalitis, encephalitis, encephalomyelitis, acute flaccid paralysis or autonomic nervous system dysregulation, pulmonary oedema, pulmonary haemorrhage, or cardiorespiratory failure), or those who died, were classified as severe HFMD cases. From January 2016 to December 2017, 12,608 patients were diagnosed with HFMD at Xinhua Hospital affiliated to Shanghai Jiao Tong University School of Medicine. The distribution of patients covered all 16 municipal districts in Shanghai.

Patients who met the following criteria were recruited in our study: 1. neonates diagnosed less than 28 days after birth; 2. skin lesions manifested as small vesicles, papulovesicular lesions or macular rashes on the palms, soles, buttocks, and oral mucosa, or were present on the limbs, trunks or facial areas. All family members were included in the screening. Since the prognosis of neonatal HFMD is unknown, they were all admitted to the hospital for observation, including routine clinical blood examination, evaluation of biochemical and immune function, and virus detection. The clinical specimens (e.g., rectal swabs and plasma) were collected from each patient within 1 day of diagnosis. To evaluate alterations in specific parameters, such as in immune function, we also recruited age- and birth weight-matched non-infected neonates (e.g., infants with breast milk jaundice) as neonatal controls, and age-matched preoperative patients without infection (e.g., subjects with hypospadias) as elder sibling controls. The control subjects had no symptoms of HFMD and tested negative for enteroviruses. Finally, 16 neonates with HFMD and their infected families were included in the study and followed up for at least 6 months for sequelae.

This study was approved by the Ethics Committee of Xinhua Hospital, affiliated to Shanghai Jiao Tong University School of Medicine (XHEC-C-2018-082), and the procedures were carried out in accordance with the Helsinki Declaration. Parents or guardians of each case or control were required to sign a written informed consent form. The relevant tests were paid by the research group.

### Data collection

Demographic data, clinical manifestations, and laboratory findings of each participant were recorded. Fever, as well as timing and distribution of skin lesions, were evaluated. The skin lesions were classified into 8 groups based on the site: perinasal, perioral, scalp, palms/soles, lower limbs, upper limbs, abdomen, and intraoral lesions.

Complete blood cell count, liver and kidney function, and the levels of myocardial enzymes, immunoglobulins, lymphocyte subsets, and cytokines were assessed in cases and controls. The immunophenotypes of peripheral blood lymphocytes (CD3, CD4, and CD8 T-cells, NK cells) were determined by flow cytometry (Becton Dickinson Immunocytometry Systems) and analysed by Cell Quest software (Becton Dickinson). The serum levels of immunoglobulin (Ig) M, IgG, and IgA were detected by turbidimetric immunoassay. ELISA (Quantikine; R&D Systems) was used for quantitative determination of the cytokines IL-1β, IL-2R, IL-6, IL-8, IL-10, and TNF-α. The assays were performed according to the manufacturer’s instructions.

The EVs were genotyped from rectal swab specimens. Viral RNA was extracted directly from the clinical specimens using a QIAamp Viral RNA Mini Kit (Qiagen, Santa Clara, CA) and stored at − 80 °C. A commercial real time RT-PCR Kit panel (Jiangsu Bioperfectus Technologies Co., Ltd., China, http://en.s-sbio.com/) was used to determine enterovirus type and subtype, including EV-A71, CV-A16, CV-A6, and CV-A10, as previously described [22, 23]. A partial VP1 gene sequence was amplified using one-step reverse transcription polymerase chain reaction (TaKaRa) with primers 292/222 as previously described [24], and the amplicons were sequenced directly. EVs were genotyped by sequence comparison by using BLAST (http://blast.ncbi.nlm.nih.gov/Blast.cgi). The sequenced DNA fragments were assembled into complete genomes using ContigExpress project in Vector NTI version 11.5. Multiple-sequence alignments were performed using the MAFFT software (http://www.ebi.ac.uk/Tools/mafft/). Phylogenetic trees were constructed by the maximum likelihood (ML) method using the MEGA version 7 software [25].

### Statistical analysis

We calculated the means and standard deviations for normally distributed variables, and the medians (interval of quartiles) for variables with skewed distribution. For pairwise comparisons, Student’s *t*-test and nonparametric tests were applied in case of normal and non-normal distributions, respectively. Frequency and percent values were calculated for categorical variables, and the *chi*-square test was used to determine the differences in these variables between neonatal and paired siblings with HFMD. Logistic analysis was applied to calculate the risk of clinical manifestations in these two groups. All statistical analyses were conducted using SPSS 17.0 software. A *p*-value < 0.01 was regarded as statistically significant.

## Results

### Epidemiological features and pathogens of neonates and elder siblings with HFMD

**Fig. 1:**
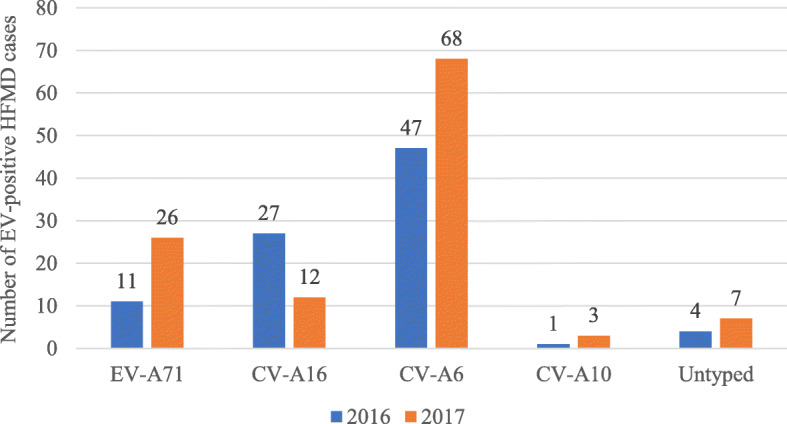
Enterovirus-positive HFMD cases, 2016–2017

Of the 12,608 HFMD cases diagnosed from 2016 to 2017 at Xinhua Hospital affiliated to Shanghai Jiao Tong University, 16 were neonates (0.13%). Among the 12,608 cases, 14 had severe HFMD, 11 of which were due to EV-A71 and 3 due to CV-A6 infection. A total of 259 patients were sampled from this sentinel hospital as part of routine EV surveillance. Of these, 206 were positive cases, with a positive rate of 79.5%. CV-A6 was the predominant causative agent of HFMD, accounting for 44.4% (115/259) of the patients (Fig. 1). In our study, all infected neonates had an elder sibling affected. Interestingly, all neonatal and elder sibling patients were infected with CV-A6. All neonatal cases were not severe, while two elder siblings developed severe HFMD. Half of the neonates with HFMD were diagnosed during summer.

### Clinical presentation

**Fig. 2:**
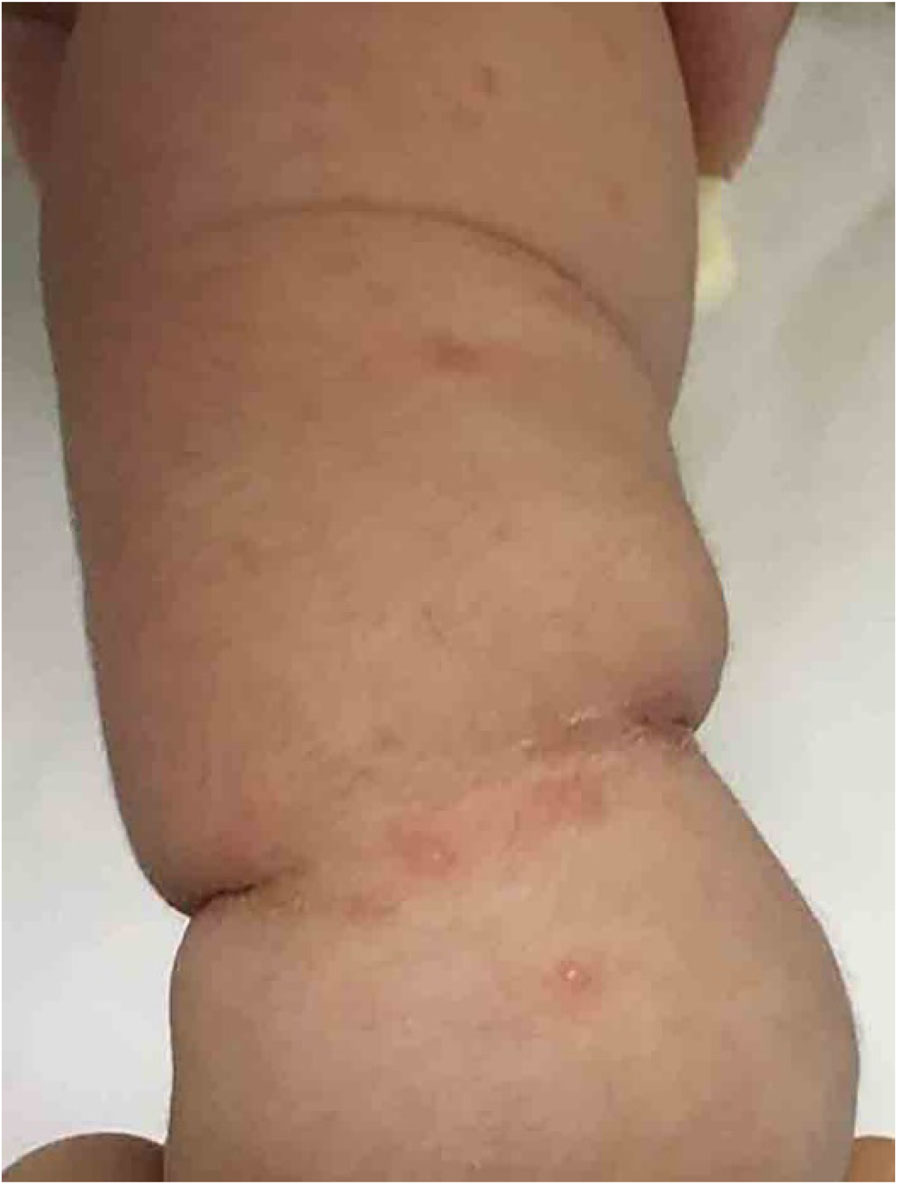
A 22-day-old boy with CV-A6 infection showing vesicles on the upper limb

The age of neonates with HFMD had a non-normal distribution, as the median value was 25 days and ranged between 19 and 28 days. All neonates were full-term with a normal birth weight. The median age of their elder siblings was 4.1 years, ranging from 2.4 to 6.3 years. The percentage of males was 37.5% among neonates and 62.5% among elder siblings. As shown in Table 1, the HFMD symptoms were milder in neonates than in their elder siblings. None of the neonates developed complications such as hypoperfusion, lethargy, onychomadesis, or others. Most of the elder siblings, but none of the neonates, had fever. Ten elder sibling patients had vomiting symptoms, two had lethargy, and one developed hypoperfusion. The prevalence of vomiting was 5 times higher among elder siblings than among neonates. Cutaneous lesions, especially intraoral erosions, were more common in elder siblings than in neonatal cases. In neonatal cases, the site of the rash was not typical, mainly involving the perioral area and the upper limbs (Fig. 2). After about 1 week of symptomatic treatment, the clinical symptoms of neonatal cases receded, and no onychomadesis or neurological sequelae occurred within half a year. However, five elder siblings had symptoms of onychomadesis 2–4 weeks after disease onset, and developed new nails within 2 months.

**Table 1:** Clinical features of neonates and paired older siblings with hand, foot, and mouth disease

Clinical features *N* (%)	Neonates (*N* = 16)	Older siblings (*N* = 16)	***Odds ratio***	***P-value***
**Fever (temperature ≥ 39 °C)**	0	14 (87.5)	0 (0.0, 0.1) ^*b^	< 0.01^*a^
**Vomiting**	2 (12.5)	10 (62.5)	0.2 (0.1, 0.8) ^*^	< 0.01^*^
**Seizure**	0	0	1.0 (0.1, 7.3) ^b^	0.50
**Symptoms of hypoperfusion**	0	1 (6.3)	0 (0.0, 19.0) ^b^	0.50 ^a^
**Lethargy**	0	2 (12.5)	0 (0.0, 3.4) ^b^	0.24
**Cutaneous areas affected**				
Perinasal	4 (25.0)	3 (18.8)	1.4 (0.2, 9.1)	0.69
Perioral	13 (81.3)	14 (87.5)	0.9 (0.7, 1.3)	0.33
Scalp	0	0	1.0 (0.1, 7.3) ^b^	0.50
Palms/soles	6 (37.5)	13 (81.3)	0.4 (0.2, 0.8) ^*^	0.02
Lower limbs	2 (12.5)	9 (56.3)	0.3 (0.1, 1.0) ^*^	0.01
Upper limbs	14 (87.5)	13 (81.3)	1.3 (0.4, 4.0)	0.66
Abdomen	0	1 (6.3)	0 (0.0, 19.0) ^b^	0.50 ^a^
Intraoral erosions	10 (62.5)	16 (100.0)	0.4 (0.2, 0.6) ^*^	< 0.01*
**Onychomadesis**	0	5 (31.3)	0 (0.0, 0.7) ^*^	0.02
**Complications**	0	1 (6.3)	0 (0.0, 19.0) ^b^	0.50 ^a^

^a^ Two-tailed exact *P*-value

^b^ Conditional maximum likelihood estimate of odds ratio

* Statistical significance

### Phylogenetic analyses of representative CV-A6 strains

**Fig. 3:**
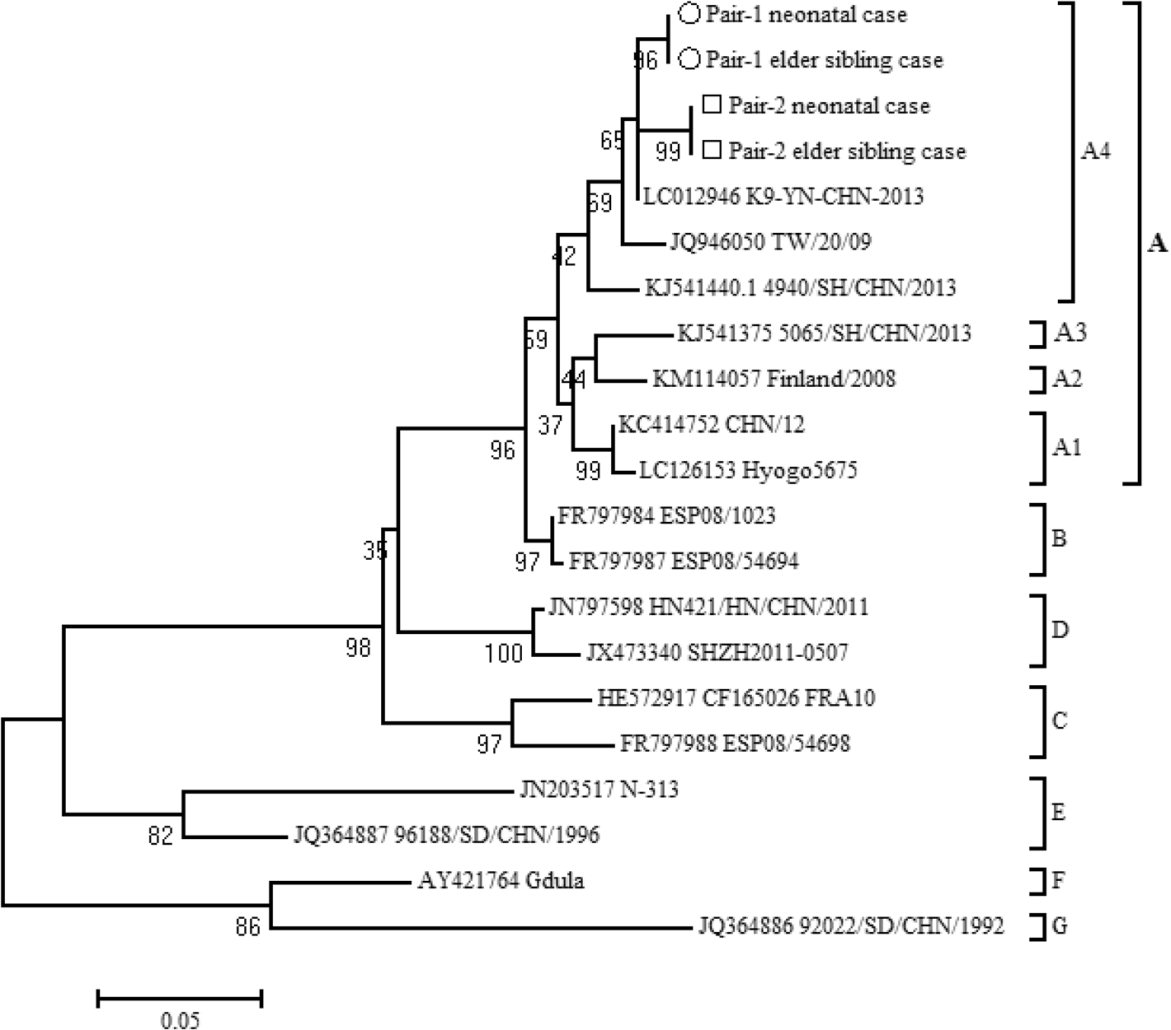
Phylogenetic analysis of CV-A6 partial VP1 nucleotide sequences. (Phylogenetic tree showing the relationships between recent clinical CV-A6 isolates from GenBank. The Genbank accession numbers and the viral isolates or strains are indicated. The scale bar indicates branch distances. Hollow circles and squares indicate the partial VP1 sequences of two representative pairs of neonatal and elder sibling cases in this study, respectively)

Partial VP1 sequences were obtained from 2 representative pair patient samples selected CV-A6-positive samples. To determine the phylogenetic relationship between Shanghai viruses and other reported CV-A6 strains, various published CV-A6 sequences were retrieved from GenBank, including the prototype Gdula isolated in 1949. Sequence similarities among strains were calculated using BioEdit 7.2.5. The partial VP1 region of 2 representative pairs sequences indicated above were deposited in GenBank under the accession numbers (MT550656-MT550659). Based on the nucleotide alignment of partial VP1 gene sequences, the highest degree of homology was observed between neonatal and elder sibling cases of two representative pairs, both with identities of 100% respectively. And nucleotide sequence similarities between the two pairs were 97.2%. A phylogenetic tree drawn on the basis of 4 representative sequences segregated CV-A6 strains into subcluster A4 (Fig. 3).

### Laboratory findings

The white blood cell (WBC) count was found to be higher in elder siblings with HFMD compared to age-matched controls (Table 2). However, such differences were not detected between neonatal cases and controls. No statistically significant differences were found in liver or kidney function, or in the levels of cardiac enzymes between cases and controls, either in neonates or their elder siblings.

**Table 2:** Routine blood test of neonates and paired older siblings with hand, foot, and mouth disease

Parameter	Neonates with HFMD	Neonate controls	***P-value***^***1***^	Older sibling with HFMD	Older sibling controls	***P-value***^***2***^	***P-value***^***3***^
**WBC count (×10**^**9**^**/L)**	8.5 (4.2, 12.8)	7.1 (5.5, 9.8)	0.51	15.5 (6.4, 20.6)	8.2 (6.7, 9.1)	< 0.01*	< 0.01*
**RBC count (× 10**^**12**^**/L)**	4.3 (3.2, 5.1)	4.1 (3.3, 4.6)	0.20	4.3 (3.5, 5.5)	4.8 (4.3, 5.4)	0.02	0.82
**Platelet count (×10**^**9**^**/L)**	378.5 (164.6, 406.9)	349.0 (248.5, 398.0)	0.01	283.1 (233.7, 369.2)	267.2 (194.2, 426.7)	0.39	0.01
**Leukomonocytes (%)**	53.4 (24.8, 76.4)	54.6 (43.7, 61.4)	0.84	47.2 (30.5, 68.3)	47.1 (41.1, 61.7)	0.92	0.78
**Monocytes (%)**	11.2 (9.4, 14.7)	9.7 (7.8, 11.7)	0.12	10.3 (7.9, 17.4)	7.4 (5.7, 9.0)	0.12	0.21
**Neutrophils (%)**	26.6 (19.2, 49.2)	22.0 (13.7, 34.2)	0.48	38.6 (13.2, 78.4)	41.7 (31.9, 50.4)	0.21	< 0.01*
**Haemoglobin (g/L)**	103.2 (89.7, 118.1)	140.0 (107.0, 154.5)	0.05	118.1 (101.3, 127.3)	127.5 (121.0, 134.0)	0.17	0.92

*WBC* White blood cells, *RBC* Red blood cells, *P-value*^*1*^*T*-test between neonatal HFMD cases and age-matched controls, *P-value*^*2*^*T*-test for the comparison between older siblings with HFMD and age-matched controls, *P-value*^*3*^*T*-test for the comparison between neonatal and older-sibling HFMD cases

*Statistical significance

Regarding the immune function, as shown in Table 3, the levels of the inflammatory markers IL-1β, IL-2R, IL-6, and TNF-α were higher in cases compared to controls in both age groups (*P* <  0.01). The levels of IgA and IgM were higher in the elder sibling patients than in the neonate cases, which may be due to age-related immunological development. In the neonates with HFMD, the Ig levels were normal, but the level of CD8 T-cells was lower compared to age-matched controls. In particular, the neonate cases exhibited a median CD8 T-cell count of 534.0 (314.2, 824.6)/μL, while in their age-matched controls a median CD8 T-cell count of 970.0 (904.5, 1150.5)/μL was detected (*P* <  0.01). There were no significant differences in other T cell types in any of the groups.

**Table 3:** Functional immune parameters in neonates and paired older siblings with hand, foot, and mouth disease

Parameter	Neonates with HFMD	Neonate controls	***P-value***^***1***^	Older siblings with HFMD	Older sibling controls	***P-value***^***2***^	***P-value***^***3***^
CD3 T-cell	2837.2 (2243.3, 3982.1)	3536.3 (3196.6, 4450.2)	0.01	2212.1 (1932.2, 2918.3)	2704.5 (2040.0, 3452.0)	0.03	0.01
CD4 T-cell	2161.6 (1845.8, 4132.7)	2488.5 (2165.5, 3379.0)	0.48	1224.5 (1074.4, 2743.7)	1373 (1041.0, 1804.0)	0.12	0.01
CD8 T-cell	534.0 (314.2, 824.6)	970.0 (904.5, 1150.5)	< 0.01*	911.7 (534.8, 1843.2)	1243.5 (998.0, 1531.0)	0.27	0.02
CD16 + CD56+ (NK cell)	250.3 (123.9, 325.4)	361.0 (239.5, 478.5)	0.72	573.9 (342.7, 1267.3)	379.5 (224.0, 1091.0)	0.02	0.79
IgG	3.2 (2.3, 4.1)	6.2 (5.2, 7.0)	0.68	9.7 (6.4, 11.6)	9.5 (8.9, 10.6)	0.12	0.09
IgA	< 0.3	< 0.3	1	2.0 (1.0, 2.6)	1.2 (0.9, 1.5)	< 0.01*	< 0.01*
IgM	0.4 (0.2, 0.6)	0.2 (0.2, 0.3)	< 0.01*^c^	2.5 (1.2, 3.2)	1.1 (0.6, 1.2)	0.05	< 0.01*
IL-1β	730.1 (384.8, 937.5)	61.9 (22.5, 71.5)	< 0.01*^c^	1019.3 (776.2, 1832.9)	88.8 (56.5, 138.2)	< 0.01*^c^	< 0.01*^c^
IL-2R	1516.4 (497.3, 2732.3)	1438.5 (1277.5, 1556.5)	< 0.01*^c^	1392.9 (476.8, 1732.2)	485.0 (394.0, 669.1)	< 0.01*^c^	0.03^c^
IL-6	32.4 (28.2, 67.3)	3.8 (3.1, 48.4)	< 0.01*^c^	16.9 (14.3, 80.9)	5.2 (2.8, 14.9)	< 0.01*^c^	0.61^c^
IL-8	63.6 (14.6, 1283.6)	110.8 (19.7, 1866.0)	< 0.01*^c^	58.2 (20.8, 1282.6)	20.1 (7.7, 321.0)	< 0.01*^c^	0.91^c^
IL-10	26.4 (14.2, 46.8)	17.5 (12.3, 53.1)	0.01^c^	79.3 (41.4, 135.4)	2.5 (1.3, 32.5)	< 0.01*^c^	< 0.01*^c^
TNF-α	15.3 (9.5, 38.2)	13.2 (11.0, 26.7)	< 0.01*^c^	18.6 (11.6, 33.3)	11.2 (8.9, 17.3)	< 0.01*^c^	0.41^c^

*P-value*^*1*^*T*-test for the comparison between neonatal HFMD and age-matched controls, *P-value*^*2*^*T*-test for the comparison between older siblings with HFMD and age-matched controls, *P-value*^*3*^*T*-test for the comparison between neonatal and older sibling HFMD cases

*Statistical significance

^c^*T*-test after log transformation

## Discussion

HFMD is one of the most recognizable viral exanthems in children and adults [26], but rarely reported in neonates. According to this study, only 0.13% of all HFMD cases were neonates in Shanghai in 2016–2017. All 16 neonates became infected from other family members, mainly their elder siblings. They were all diagnosed with CV-A6 infection and had mild clinical symptoms. Neonatal HFMD cases showed normal immune function. Almost all cytokines exhibited higher plasma levels in cases than in controls.

In this study, the age of neonatal onset ranged between 19 and 28 days, and the mothers had no prenatal infection symptoms; therefore, vertical transmission was not considered. In China, mothers usually rest indoors for one full month after giving birth, avoiding contact with people outside of the family. Therefore, the chances of infection are relatively low for mothers. With the adoption of the two-child policy, the risk of infection is very high for elder siblings, who are generally pre-schoolers in kindergartens [27]. In addition, according to epidemiological evidence, elder siblings were infected earlier than the neonates, and the nucleotide sequence of CV-A6 viruse similarities between the neonates and elder siblings were 100%, which indicated that the neonates and elder siblings were infected by the same CV-A6 strain. The most likely scenario is that the elder siblings with HFMD acquired the infection from an unknown common source and transmitted the virus to neonates on returning home. This further supported within-family transmission. However, establishing the transmission pathway is still a difficult challenge.

In the literature, significant clinical differences were reported in HFMD manifestations depending on the pathogen. Genetic typing to establish the exact virus strain is usually not necessary to confirm the HFMD diagnosis. However, in some cases of HFMD, identification of the virus type is crucial for appropriate disease management and to reliably assess the risk of potential complications. The sole published case of neonatal EV-A71 infection was quite severe. Another reported case of CV-B3 infection, which was not fatal and self-limited in children, also caused severe disease in a neonatal case. However, none of the five neonates clinically diagnosed with HFMD in southeast China developed brainstem encephalitis or pulmonary oedema, and all recovered well. In our study, CV-A6 was the predominant causative agent of HFMD in all patients. Neonatal HFMD cases were all diagnosed with CV-A6 infection and exhibited mild symptoms. Moreover, the incidence of fever, vomiting, and onychomadesis was lower among neonatal cases compared to elder children. Some HFMD cases exhibited an atypical skin presentation with facial involvement and vesiculobullous lesions throughout the body.

Immunological reactions may be critical for HFMD. Almost all fatal HFMD cases had symptoms of autonomic nervous system dysregulation and increased sympathetic discharge, indicating the involvement of reticular formation [28]. Systemic inflammatory response also played an important role. Consistently, several studies have reported that virus infection activates the host immune system, causing the release of cytokines, as well as tissue and cell damage [29]. In our study, cytokine and WBC levels were increased in both neonatal and elder sibling patients. However, neonatal HFMD cases showed significantly lower CD8 T-cell counts compared to diseased elder siblings, which is not uncommon in acute viral infection. The T-cell subset assay is an accurate method to evaluate cellular immunity, and abnormal results may indicate the occurrence or aggravation of viral diseases. Notably, Wang and colleagues previously found that CD4 T-cells, CD8 T-cells, and NK cells are depleted in patients with pulmonary oedema, possibly resulting in impaired EV-A71 clearance [30]. Another study reported that CD4 T-cells are decreased, while CD8 T-cells are not affected, in patients with HFMD [31]. There are few reports on neonatal HFMD. We speculate that the low level of CD8 T-cells in neonatal cases is related to the sampling time. Although all samples were collected at admission, the time interval from onset to clinical evaluation may have differed between patients. Since only the symptomatic population was considered, this study may contain a selection bias. In addition, some results, such as the low level of CD8 T cells observed in neonatal HFMD cases, need to be confirmed by performing further studies.

## Conclusions

Neonatal HFMD caused by CV-A6 was characterized by mild clinical symptoms and basically normal immune function. Neonatal HFMD is not always a serious condition, and disease severity may depend on the pathogen. In China, with the gradual adoption of the two-child policy, elder brothers or sisters are the main source of infection. In case of infection, control measures should be in place. In addition, as the prevalence of CV-A6 is on the rise, it will be crucial to explore the suitability of CV-A6 as the main component of combined or multivalent vaccines for HFMD prevention and control.

## Data Availability

The datasets used and/or analysed during the current study are available from the corresponding author on reasonable request.

## Abbreviations

CV: Coxsackievirus
EV: Enterovirus
HFMD: Hand foot and mouth disease
WBC: White blood cell
